# Metabolomics dataset of underutilized Indonesian fruits; rambai (*Baccaurea motleyana*), nangkadak (*Artocarpus nangkadak*), rambutan (*Nephelium lappaceum*) and Sidempuan salak (*Salacca sumatrana*) using GCMS and LCMS

**DOI:** 10.1016/j.dib.2019.103706

**Published:** 2019-03-06

**Authors:** Hafidz R. Halim, Dhika P. Hapsari, Ahmad Junaedi, Arya W. Ritonga, Azis Natawijaya, Roedhy Poerwanto, Sobir, Winarso D. Widodo, Deden D. Matra

**Affiliations:** a Department of Agronomy and Horticulture, Faculty of Agriculture, IPB University (Bogor Agricultural University), Indonesia; b Research and Development Division, Mekarsari Fruit Garden, Indonesia

## Abstract

The information of secondary metabolite compound from underutilized Indonesian fruits are still limited including rambai (*Baccaurea motleyana* Müll.Arg.), nangkadak (*Artocarpus nangkadak* or *A. heterophyllus* x *A. integer*), rambutan (*Nephelium lappaceum* L.) and Sidempuan salak (*Salacca sumatrana* Becc.). To identify the secondary metabolite, we used GC-MS (gas chromatography-mass spectrometry) and LC-MS (liquid chromatography-mass spectrometry) analyses. The accessions/varieties numbers used in this analysis including two accession for rambai, three accessions for nangkadak, four varieties for rambutan and three accessions for Sidempuan salak. All sample were collected from edible part such arilode/carpel and also rind for only rambutan. Based on, spectral data showed common and specific secondary metabolite compounds in each commodity. Preliminary GCMS analysis from the dataset obtained specific secondary metabolites contained in rambai; Decanoic acid, 1-Decene, Methyl salicylate and Stearyl alcohol, nangkadak; β-Cyclocitral, 2-Furanmethanol and Linoleic acid, rambutan; Citraconic anhydride, 3,5-Dideuteropyridine-4-carboxylic acid, Isobutyl formate and n-Methyl-D3-Aziridine, and Sidempuan salak; 5-Formyl-2-furfurylmethanoate, 2-Methoxy-4-vinylphenol and Tiglic acid.

**Table:** Specifications table

Subject area	Agricultural and Biological Sciences
More specific subject area	Horticulture
Type of data	Raw data and Excel Table
How data was acquired	Metabolomics data were obtain using GC-MS Agilent Technologies 7890A/G3440A 5975C inert/G3171A and LC-MS Waters 2695 Quattro Micro MSMS QAA 842
Data format	Raw data (compressed zip) and filtered (xlsx MS Excel format)
Experimental factors	The sample was collected from arilode/carpel/rind at mature stage
Experimental features	Ethyl acetate extract for GCMS analysis and ethanol extract for LCMS analysis
Data source location	Cileungsi, Bogor, West Java, Indonesia (6°24′50.1″S 106°59′05.7"E)
Data accessibility	- Raw data available at https://doi.org/10.17632/k4h8h86gwt.1 and https://rujakbase.agrohort.ipb.ac.id/ - Supplementary Table 1
Related research article	D. D. Matra, A. W. Ritonga, A. Natawijaya, R. Poerwanto, Sobir, W. D. Widodo, E. Inoue, Dataset of the first de novo transcriptome assembly of the arillode of Baccaurea motleyana, Data in Brief, (2019) 332-335 D. D. Matra, A. W. Ritonga, A. Natawijaya, R. Poerwanto, Sobir, W. D. Widodo, E. Inoue, Dataset from de novo transcriptome assembly of Nephelium lappaceum aril, Data in Brief Elsevier, 22 (2019) 566-569

**Table:** 

**Value of the data** • The GCMS and LCMS of Indonesian underutilized fruits datasets provides secondary metabolite profiling to identify specific compound among accession and variety for further characterization • The metabolomic data provide many information to improve fruit quality and nutritional value related to postharvest technology • The raw data available in public repository that could be reused for further analysis

## 1 Data

In this study, four underutilized Indonesian fruits of metabolomic data were analyzed using GCMS and LCMS-based techniques even the transcriptomic data from rambai [1] and rambutan [2] have been available. Rambai is one of the species from Baccaurea genus that have a volatile compound such as (E)-Hex-2-enal and methyl-2-hydroxy-3-methylbutanoate as an antibacterial activity [3]. Nangkadak is a cross hybridization between Jackfruit var. nangka mini (*Artocarpus heterophyllus*) and cempedak (*Artocarpus integer*) in which for the first time introduced as “Mekarsari” variety in Mekarsari Fruit Garden [4]. Rambutan already has several released varieties, such as Binjai, Sinyonya, Lebak Bulus, Rapiah, and Cimacan. Rambutan also has some antioxidant properties, such as anthocyanin, phenolic and methanolic [5]. Sidempuan salak fruit has various types of aril or flesh color, such as white, reddish white and red dominant [6]. This fruit may contain rich of flavonoid, glycoside, saponin, tannin, epicatechin, proanthocyanidin [7] and steroid [8] as an antioxidant.

## 2 Experimental design, materials, and methods

The plant materials used two accessions of rambai (white and red arilodes), three accessions of Nangkadak (Super Orange, Jumbo, and Bola), four varieties of rambutan (Binjai, Cikoneng, Rapiah, Sinyonya), and three accesions of Sidempuan salak (white, reddish, and dominant red of aril). The fruit samples were collected from Mekarsari Fruit Garden (Cileungsi, Bogor, West Java, Indonesia), then the preparation of sample was conducted at Postharvest Laboratory (Department of Agronomy and Horticulture, IPB University, Indonesia). Each fruit was separated carefully into three parts including rind, aril (fruit flesh), and seeds. The aril from all fruits and rind parts for only rambutan were separated from the seeds and then collected into a conical tube at −20 °C freezer until further analysis. The sample was dried in the oven approximately 3 hours. The dried sample was blended then macerated with ethyl acetate for GC-MS and ethanol for LC-MS analysis. The macerated sample was put into the new tube and then dried for 1 hour at 60 °C. Then, they resolved again with 200 μl maceration extract. The sample was performed for analysis using Agilent Technologies 7890A/G3440A 5975C inert/G3171A for GC-MS and Waters 2695 Quattro Micro MSMS QAA 842 for LC-MS. The raw dataset was collected and analyzed using AnalyzerPro Software (SpectralWorks Ltd). The filtered data from all samples were collected in MS Excel Format.

## 3 Data accessibility

All raw data have been deposited to Mendeley Data at https://doi.org/10.17632/k4h8h86gwt.1 [9] and https://rujakbase.agrohort.ipb.ac.id.
